# Supplementary material for: New Flavonoid Derivatives from Melodorum fruticosum and Their α-Glucosidase Inhibitory and Cytotoxic Activities
Source: Molecules. 2022 Jun 22;27(13):4023. doi: 10.3390/molecules27134023 (PMC9268484; doi:10.3390/molecules27134023)
Supplement: Supplementary file 1 [file molecules-27-04023-s001.zip › molecules-1776789-supplementary.pdf]

# Supplementary Materials

## New Flavonoid Derivatives from *Melodorum fruticosum* and Their $\alpha$-Glucosidase Inhibitory and Cytotoxic Activities

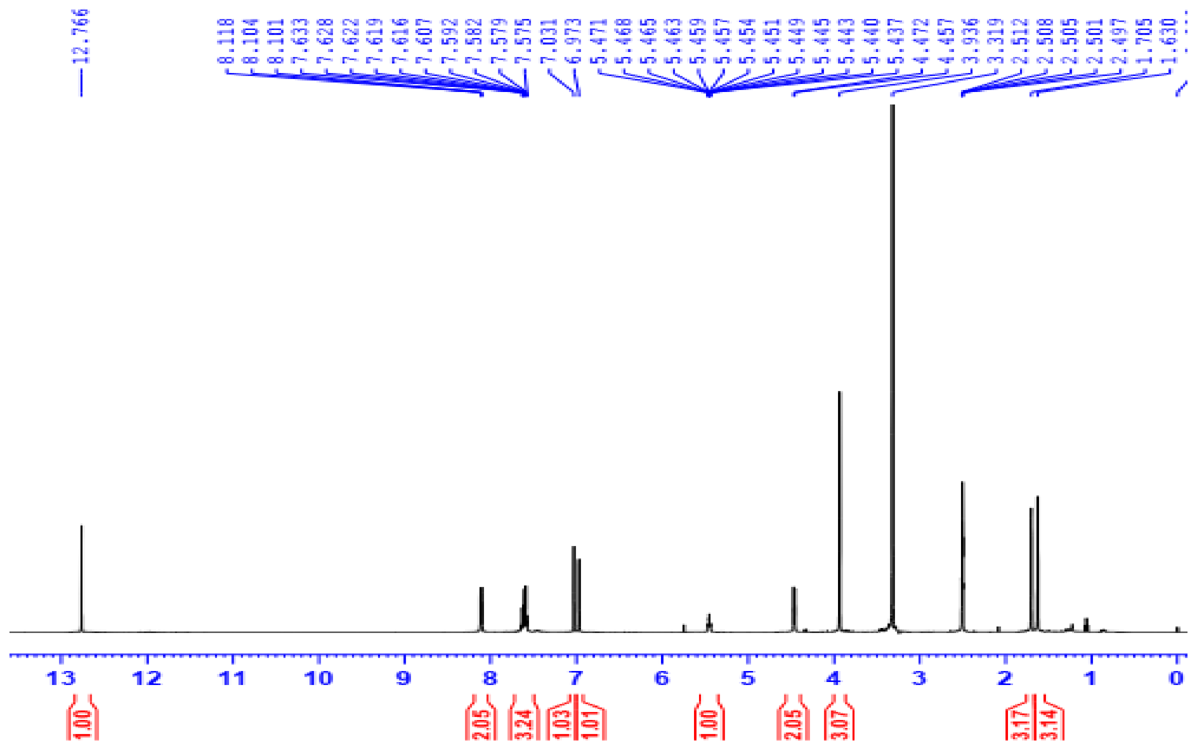


**Figure S1.** The $^{1}$H NMR spectrum of **1** in DMSO-$d_6$

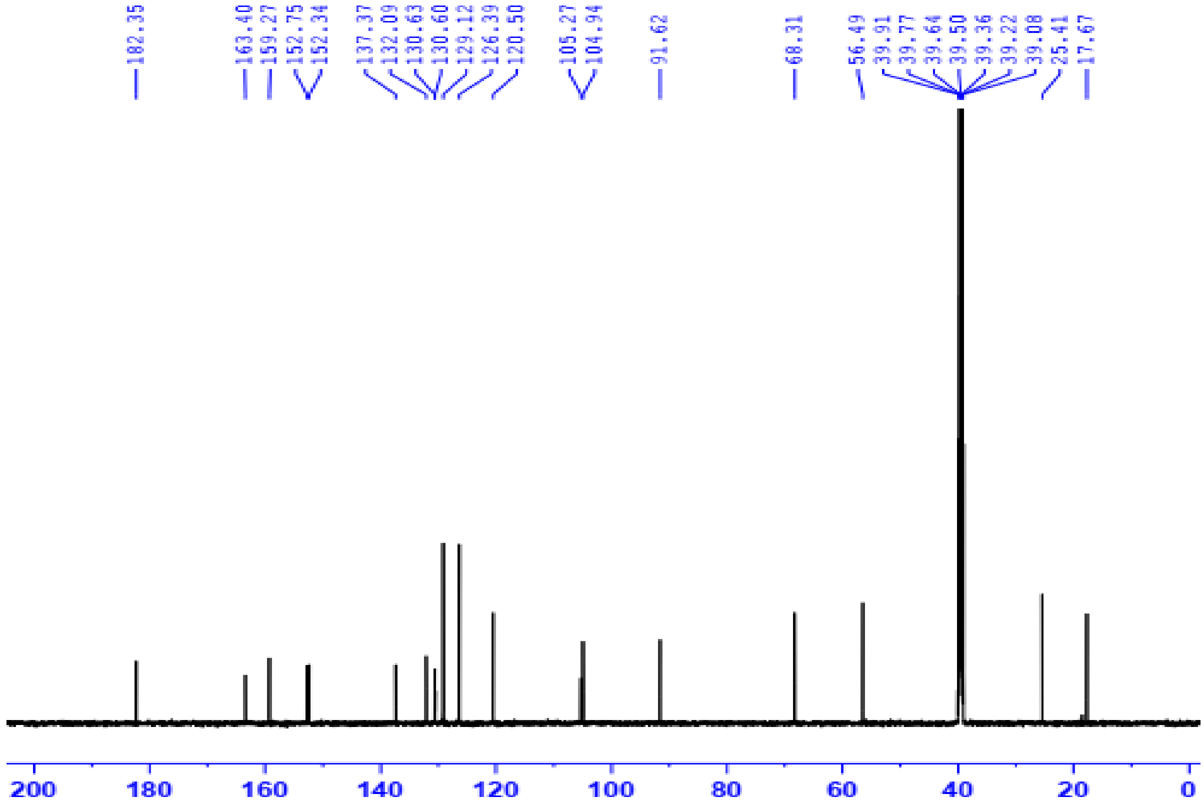


**Figure S2.** The $^{13}$C NMR spectrum of **1** in DMSO-$d_6$

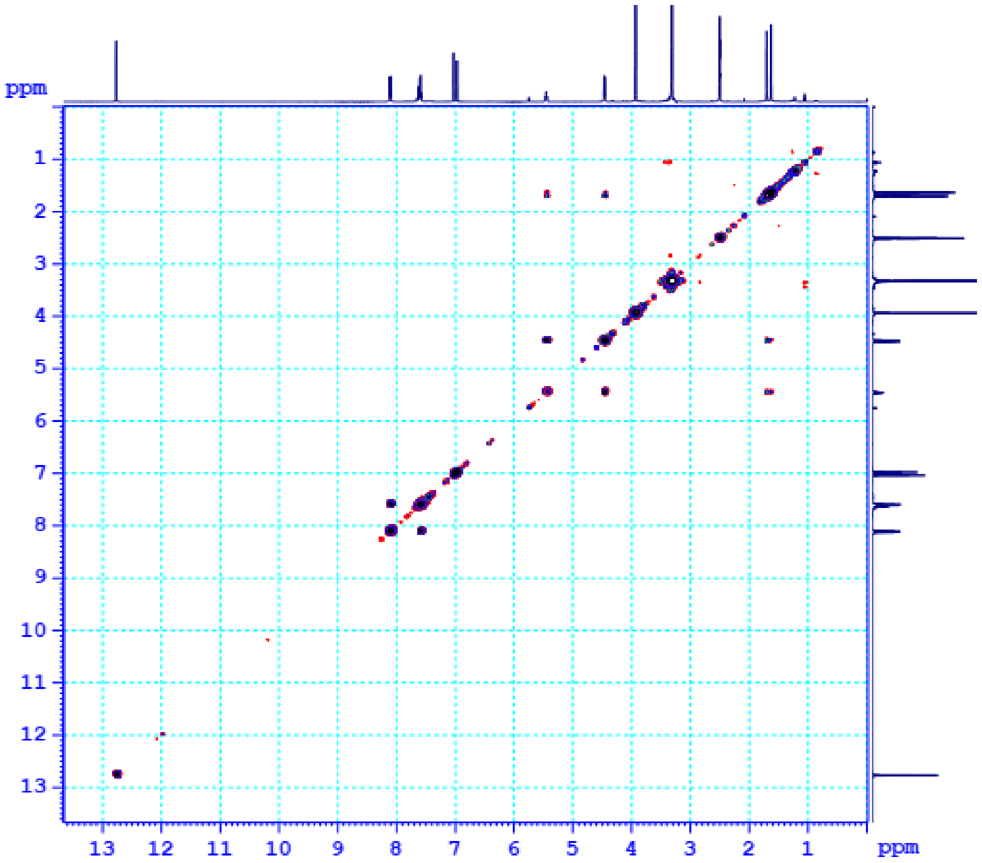


**Figure S3.** The COSY spectrum of **1**

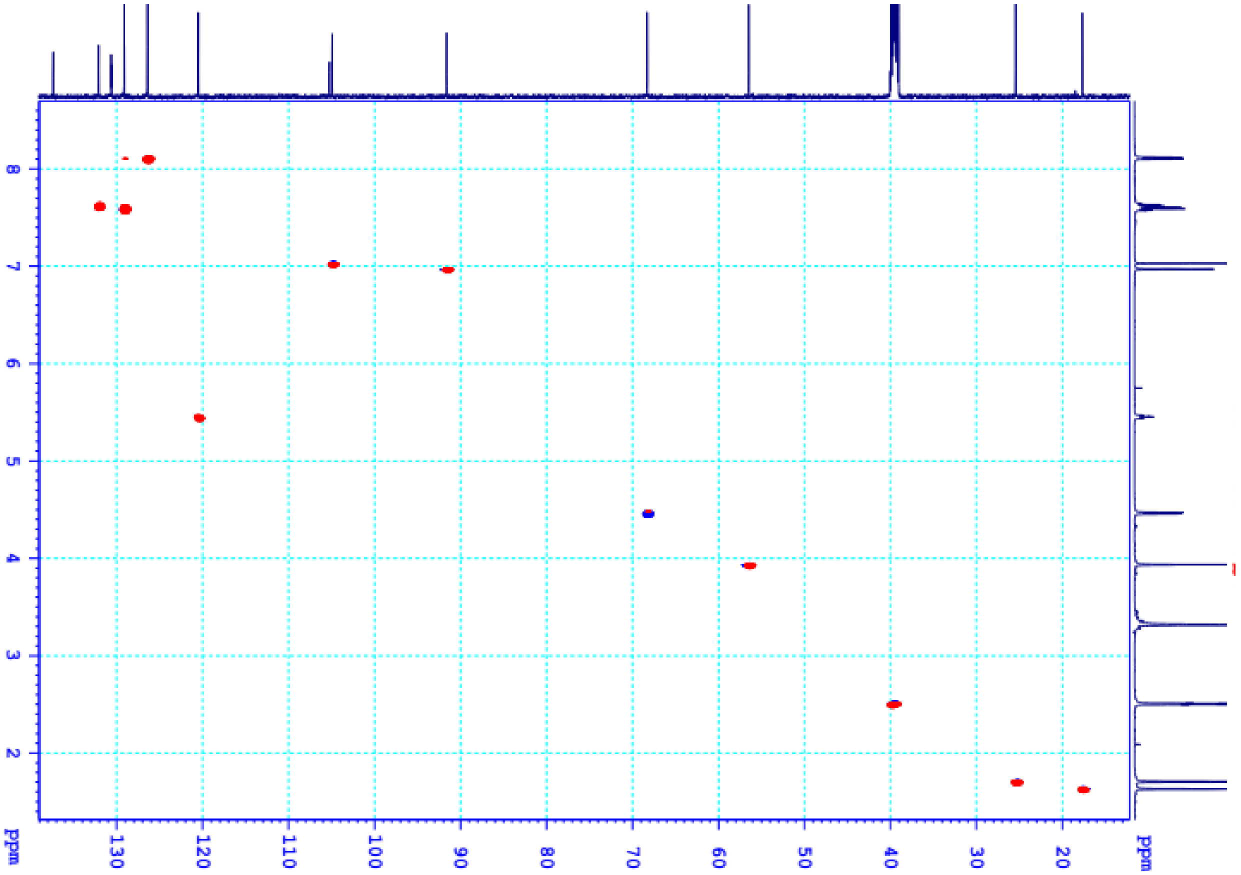


**Figure S4.** The HSQC spectrum of **1**

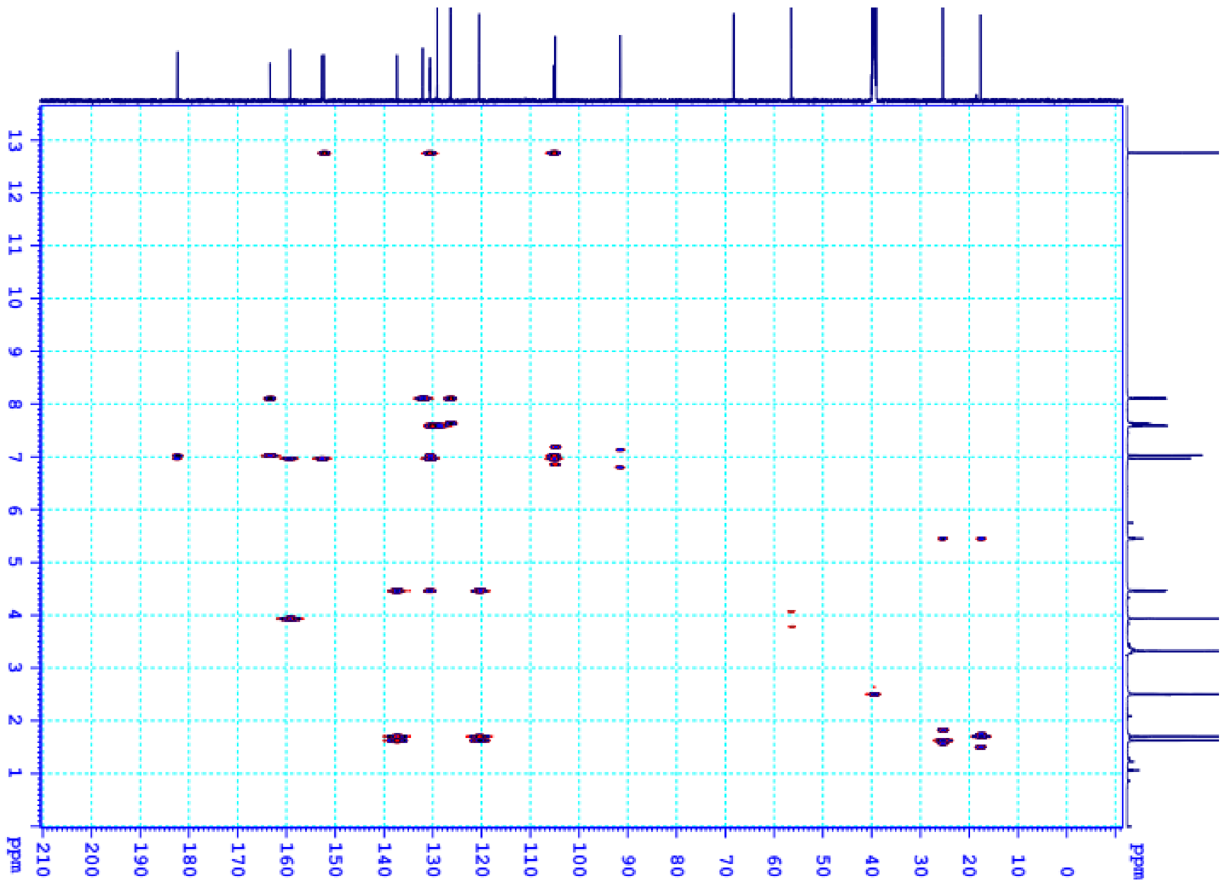


**Figure S5.** The HMBC spectrum of **1**

Created with SCIEX OS 1.2

**CENTER FOR RESEARCH AND TECHNOLOGY TRANSFER**

**PHARMACEUTICAL CHEMISTRY LABORATORY**

*1B, Thanh Loc 29 St., Dist 12, Ho Chi Minh City, Vietnam. Phone: (84) 907 070 939*

---

## ANALYSIS REPORT

**Injection details**

| | | | |
|---|---|---|---|
| *Sample name* | LIEN 166 | *Vial position* | 21 |
| *Sample file name* | SER. wiff2 – LIEN | *Inject volume* | 5.00 |
| *Acquisition date* | 18/05/2021 16:06:23 PM | *Acquisition method* | ESI _NEG_SCAN |
| *Operator* | CB21261708 | *Instrument name* | X500R QTOF |

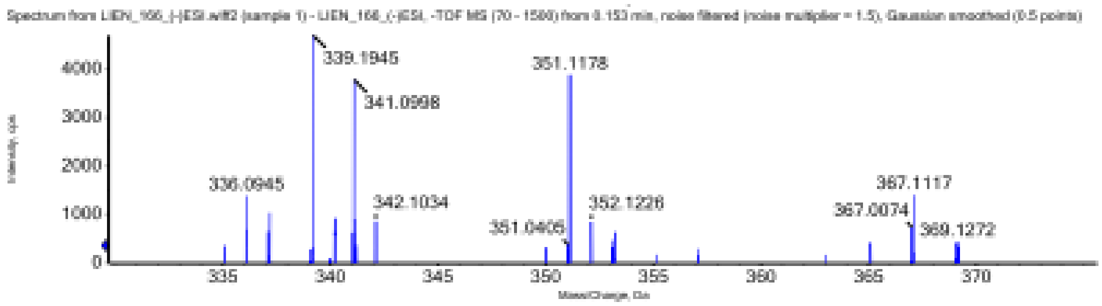


**Figure S6.** The HRESIMS spectrum of **1**

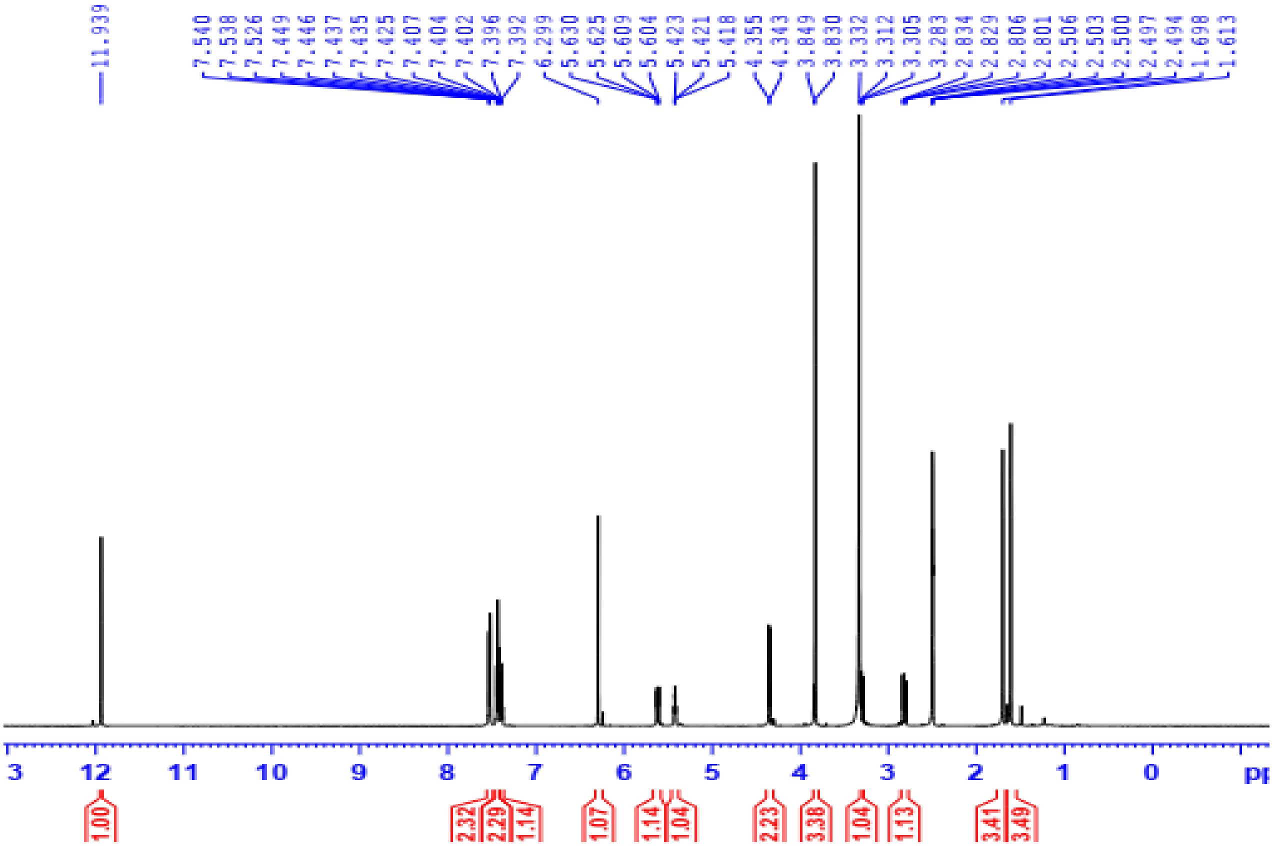


**Figure S7.** The $^{1}H$ NMR spectrum of **2** in DMSO-$d_6$

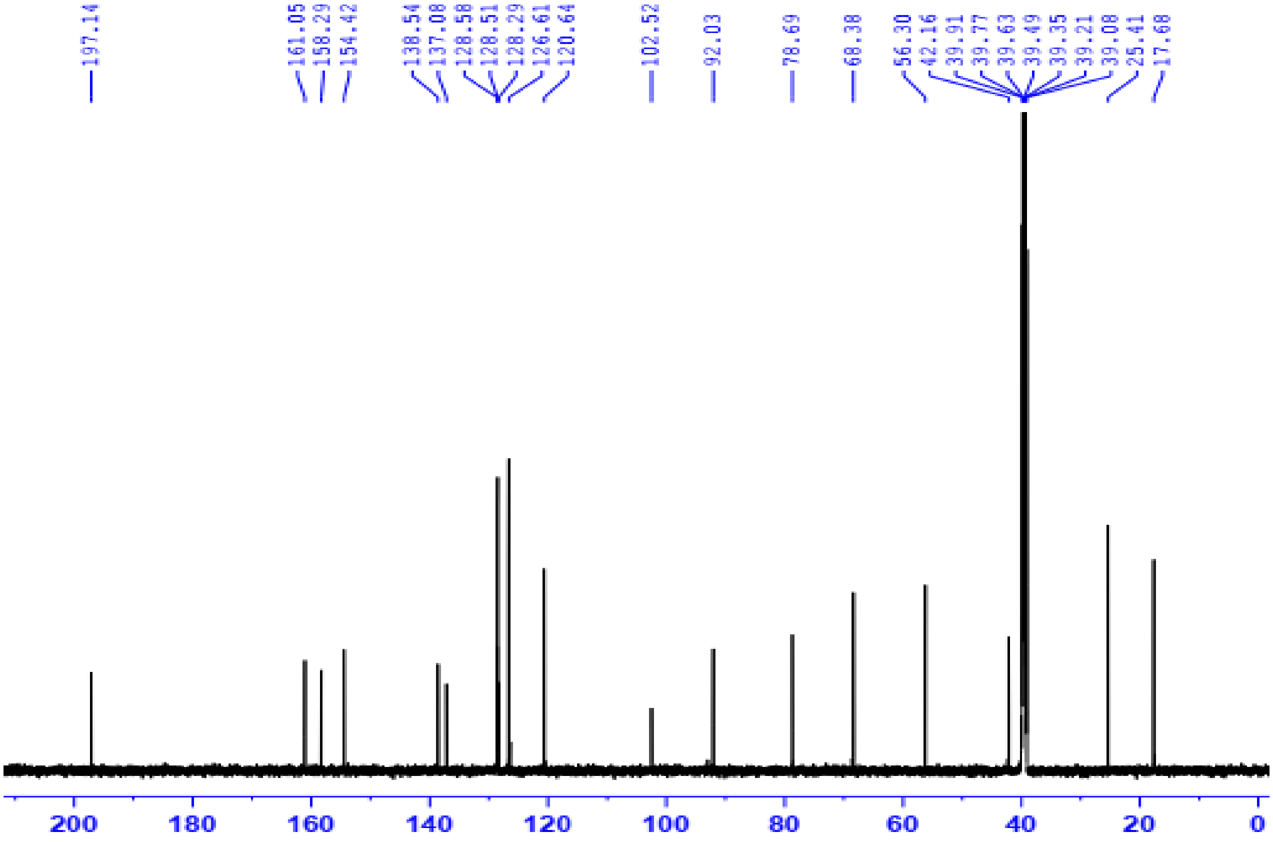


**Figure S8.** The $^{13}C$ NMR spectrum of **2** in DMSO-$d_6$

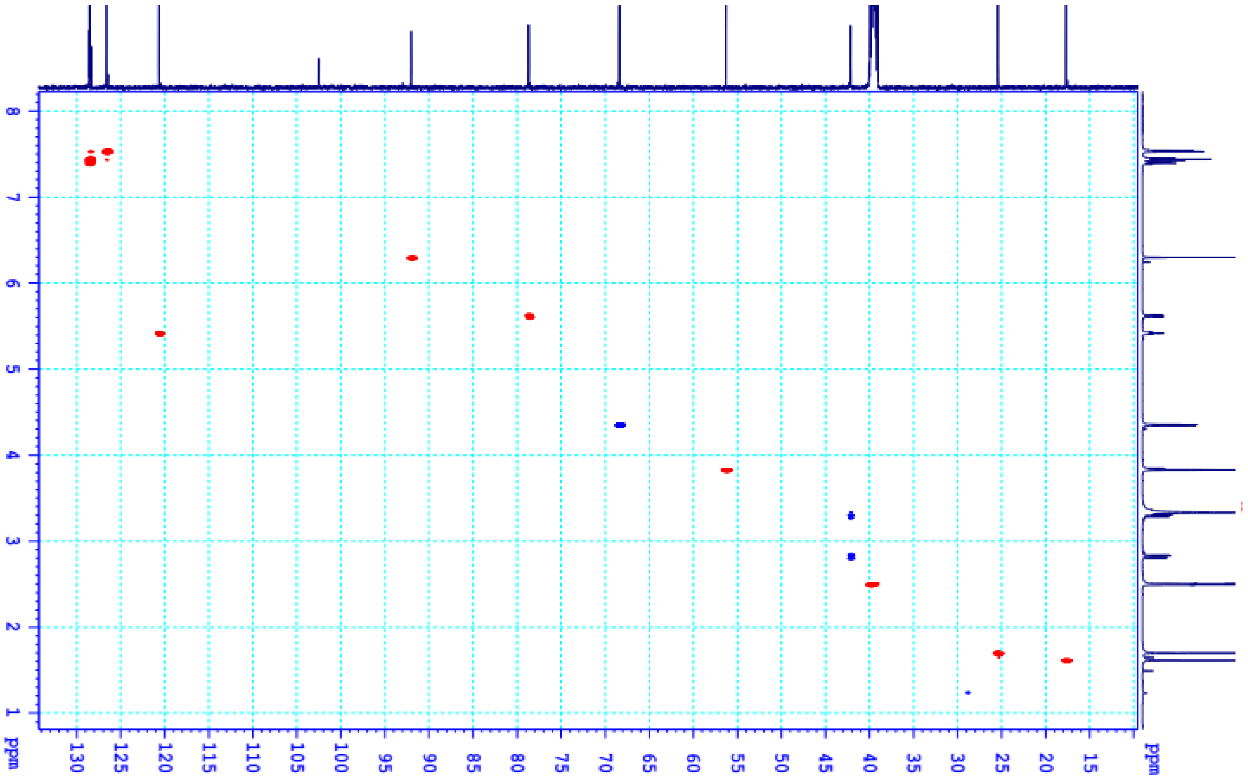


**Figure S9.** The HSQC spectrum of **2**

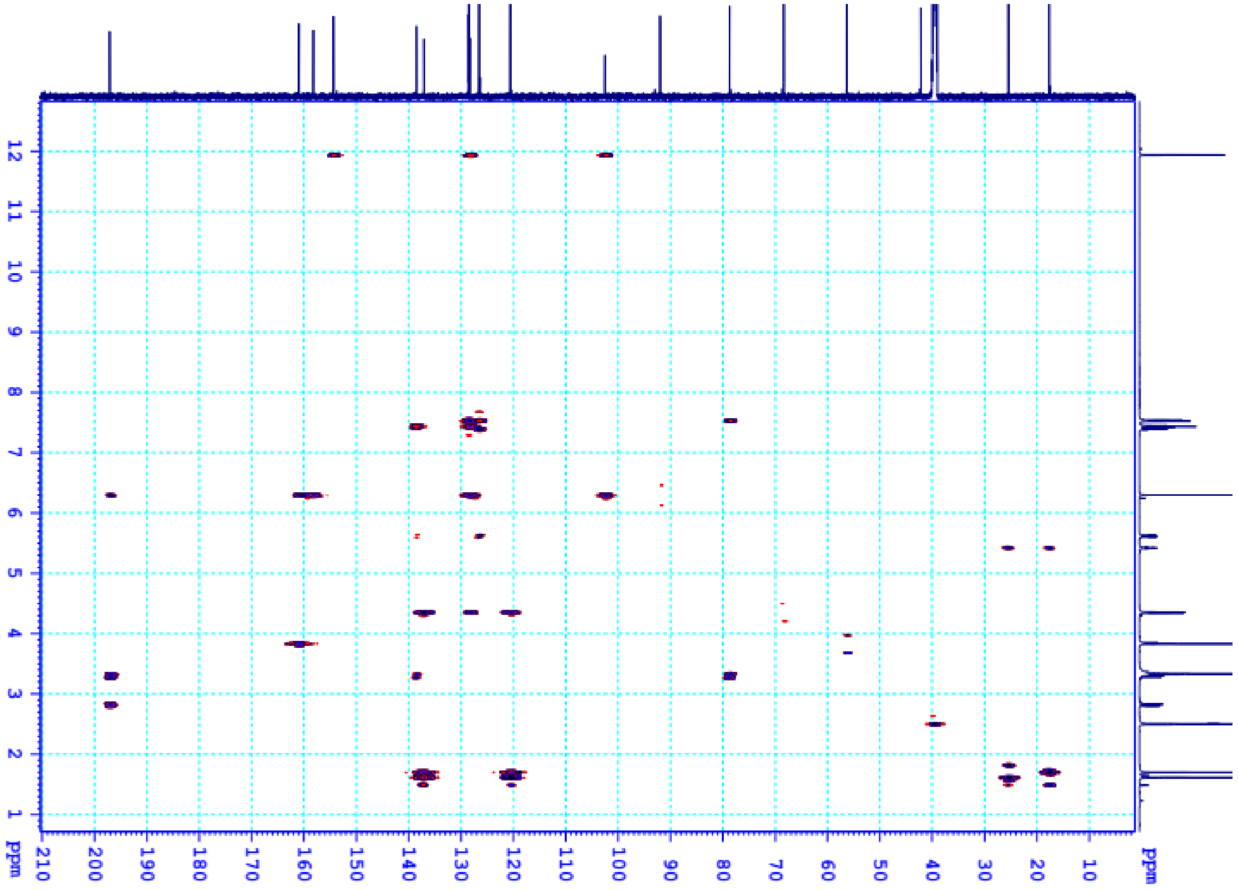


**Figure S10.** The HMBC spectrum of **2**

# ANALYSIS REPORT

## Injection details

| | | | |
|---|---|---|---|
| *Sample name* | LIEN_157 | *Vial position* | 6 |
| *Sample file name* | SER. wiff2-LIEN | *Inject volume* | 5.00 |
| *Acquisition date* | 07/05/2022 11:10:02 AM | *Acquisition method* | **ESI _NEG_SCAN** |
| *Operator* | CB21261708 | *Instrument name* | X500R QTOF |

## Full mass spectrum

Spectrum from LIEN 157_(-)ESI 2022-05-07-11-10-02.wiff2 (sample 1) - LIEN 157_(-)ESI, -...from 0.171 min, noise filtered (noise multiplier = 1.5), Gaussian smoothed (0.5 points)

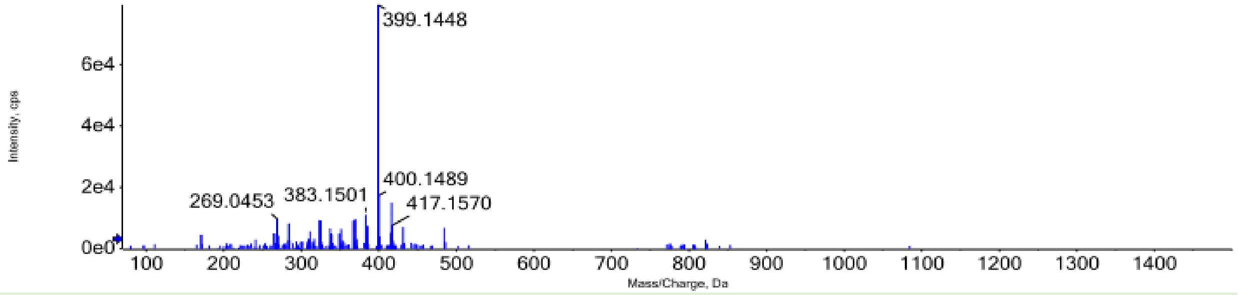


## Expanded spectrum

Spectrum from LIEN 157_(-)ESI 2022-05-07-11-10-02.wiff2 (sample 1) - LIEN 157_(-)ESI, -...from 0.171 min, noise filtered (noise multiplier = 1.5), Gaussian smoothed (0.5 points)

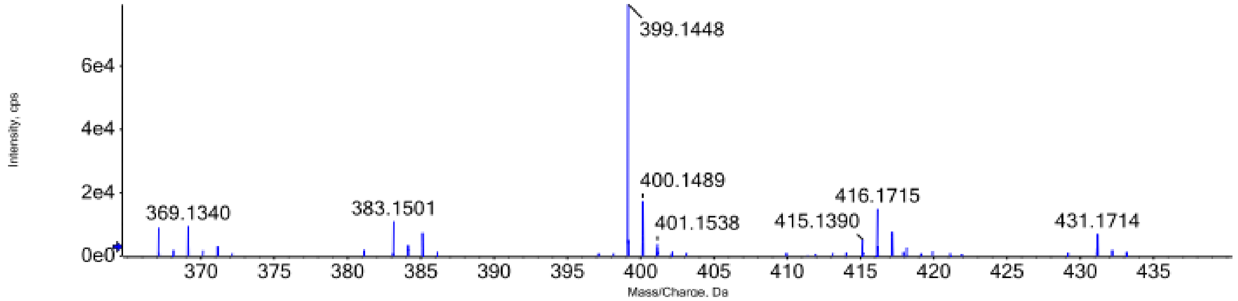


**Figure S11.** The HRESIMS spectrum of **2**

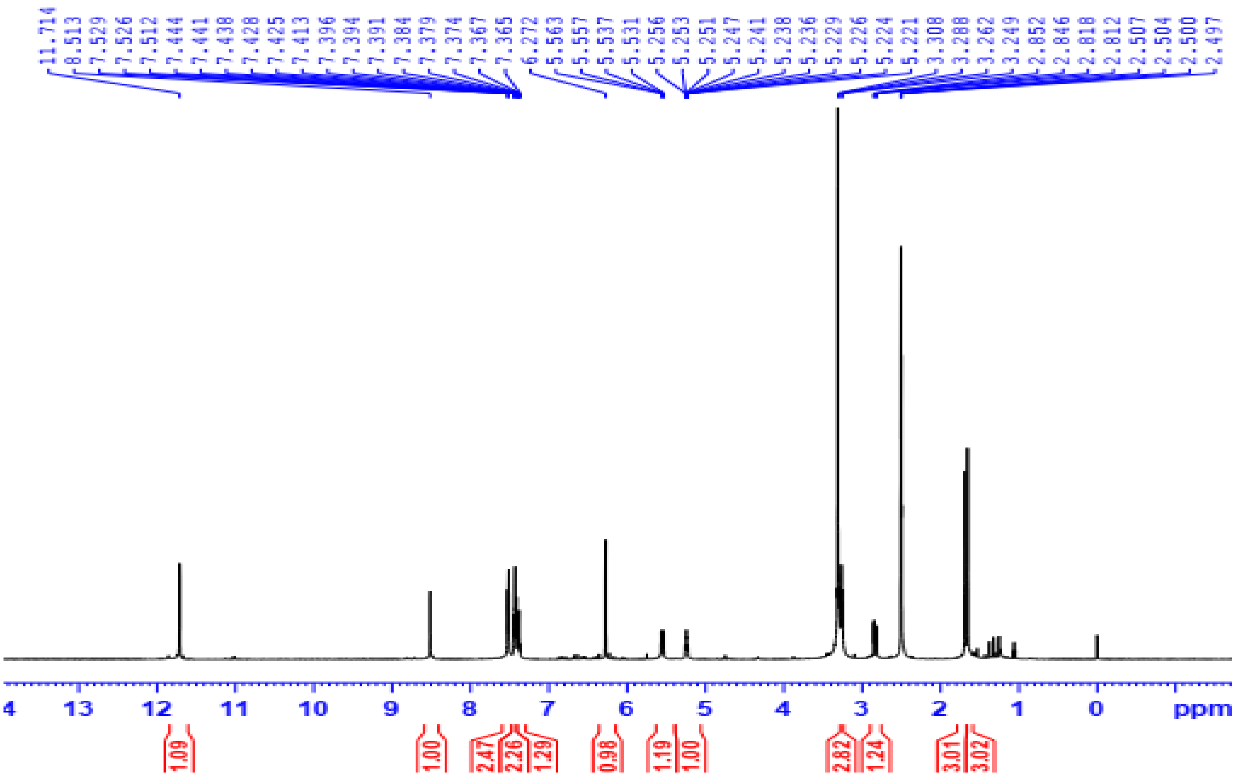


**Figure S12.** The $^{1}$H NMR spectrum of **3** in DMSO-$d_6$

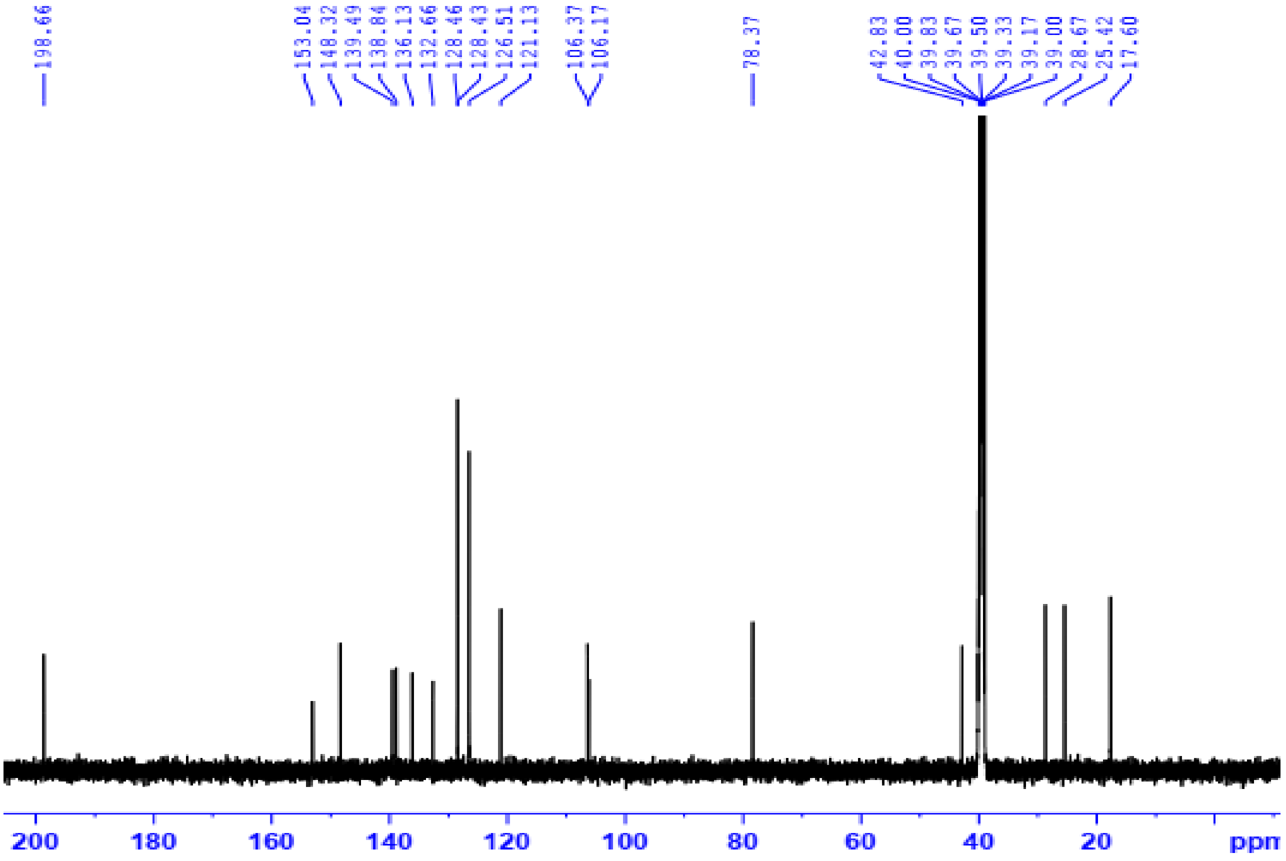


**Figure S13.** The $^{13}$C NMR spectrum of **3** in DMSO-$d_6$

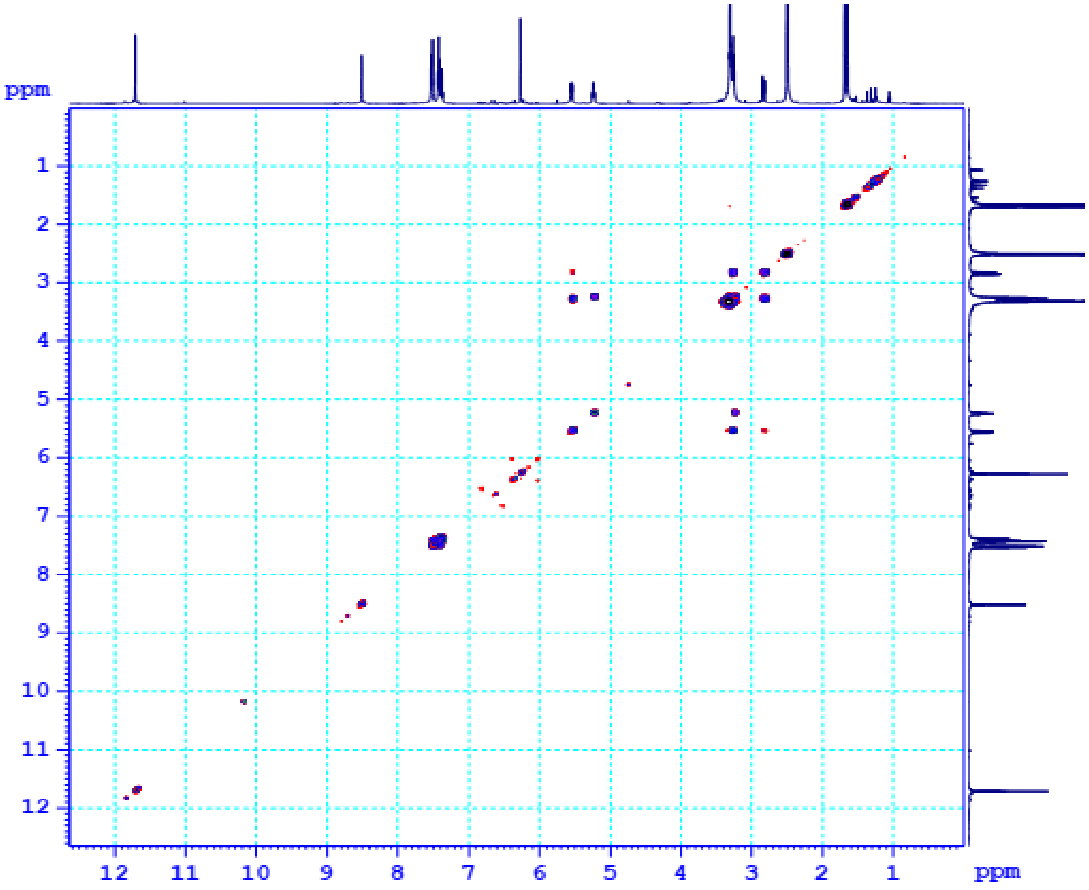


**Figure S14.** The COSY spectrum of **3**

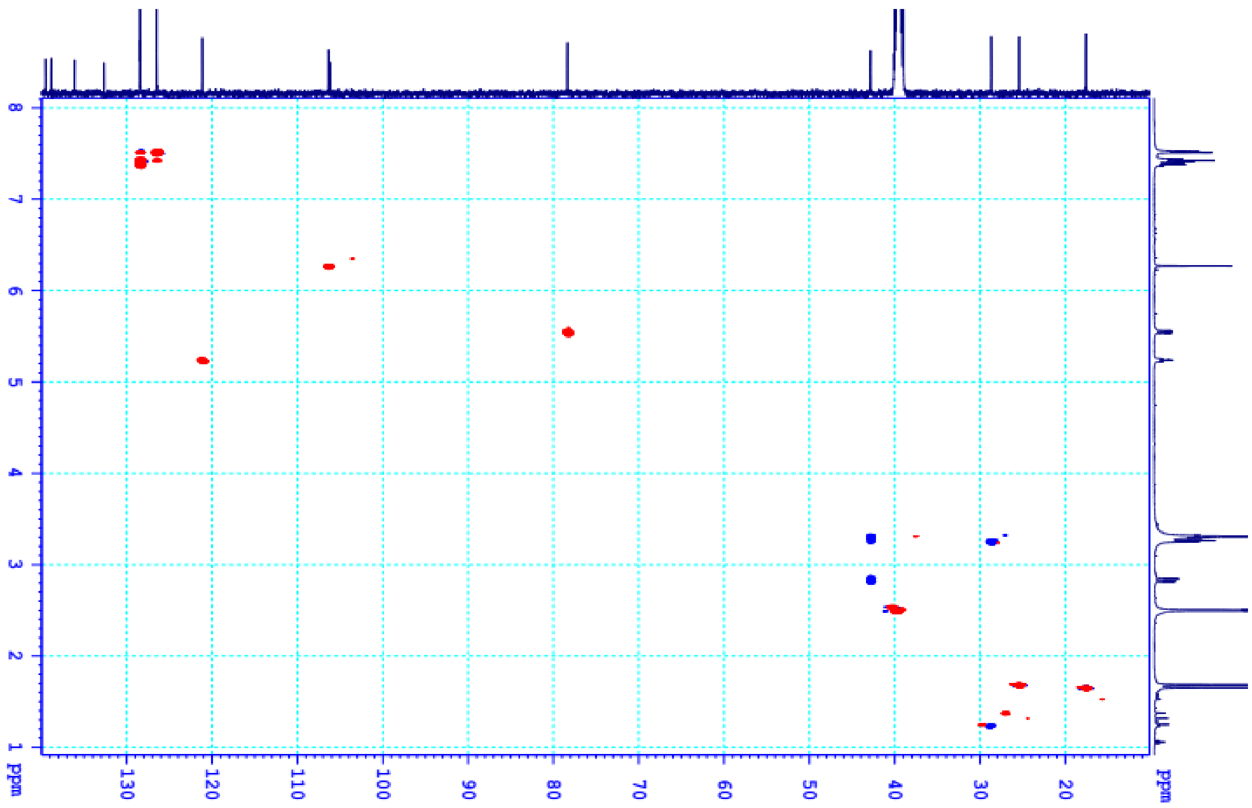

**Figure S15.** The HSQC spectrum of **3**

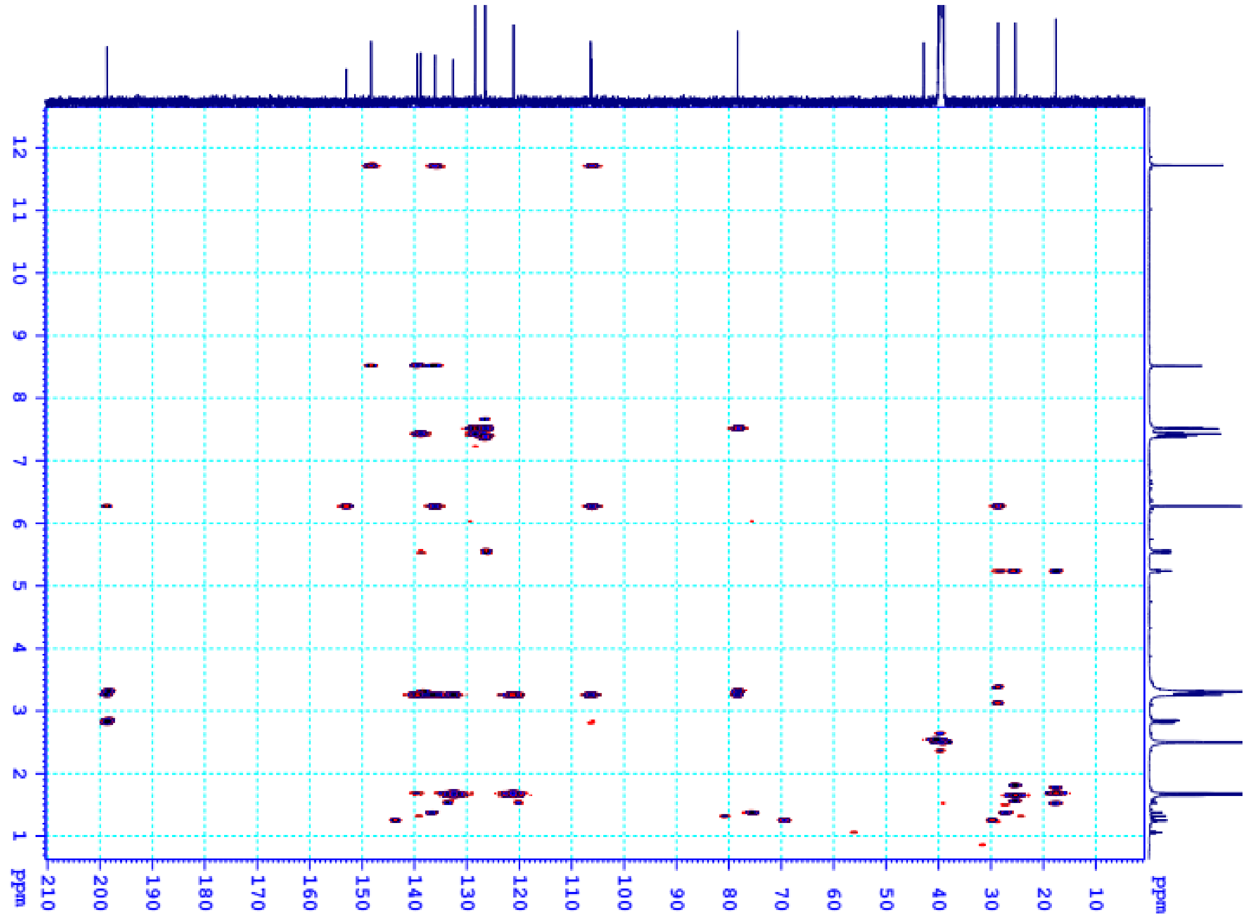

**Figure S16.** The HMBC spectrum of **3**

Created with SCIEX OS 1.2

CENTER FOR RESEARCH AND TECHNOLOGY TRANSFER
PHARMACEUTICAL CHEMISTRY LABORATORY
1B, Thanh Loc 29 St., Dist 12, Ho Chi Minh City, Vietnam. Phone: (84) 907 070 939

# ANALYSIS REPORT

## Injection details

| | | | |
|---|---|---|---|
| *Sample name* | LIEN 162 | *Vial position* | 16 |
| *Sample file name* | SER. wiff2 – LIEN | *Inject volume* | 5.00 |
| *Acquisition date* | 18/05/2021 16:02:23 PM | *Acquisition method* | **ESI _NEG_SCAN** |
| *Operator* | CB21261708 | *Instrument name* | $X500_R$ QTOF |

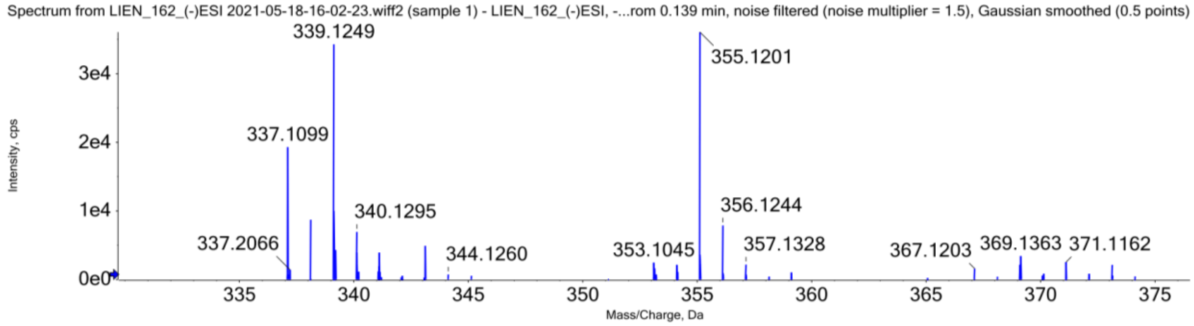


**Figure S17.** The HRESIMS spectrum of **3**
